# Swiss Army Pathogen: The *Salmonella* Entry Toolkit

**DOI:** 10.3389/fcimb.2017.00348

**Published:** 2017-08-09

**Authors:** Peter J. Hume, Vikash Singh, Anthony C. Davidson, Vassilis Koronakis

**Affiliations:** Department of Pathology, University of Cambridge Cambridge, United Kingdom

**Keywords:** *Salmonella* invasion, T3SS effectors, membrane ruffling, actin cytoskeleton, *Salmonella* pathogenicity islands, SPI1-independent entry

## Abstract

*Salmonella* causes disease in humans and animals ranging from mild self-limiting gastroenteritis to potentially life-threatening typhoid fever. Salmonellosis remains a considerable cause of morbidity and mortality globally, and hence imposes a huge socio-economic burden worldwide. A key property of all pathogenic *Salmonella* strains is the ability to invade non-phagocytic host cells. The major determinant of this invasiveness is a Type 3 Secretion System (T3SS), a molecular syringe that injects virulence effector proteins directly into target host cells. These effectors cooperatively manipulate multiple host cell signaling pathways to drive pathogen internalization. *Salmonella* does not only rely on these injected effectors, but also uses several other T3SS-independent mechanisms to gain entry into host cells. This review summarizes our current understanding of the methods used by *Salmonella* for cell invasion, with a focus on the host signaling networks that must be coordinately exploited for the pathogen to achieve its goal.

## Introduction

*Salmonella enterica* is a leading cause of morbidity and mortality in both humans and animals. Different serovars have distinct species specificity as well as diverse clinical manifestations (Gal-Mor et al., 2014). For example, the human-specific *Salmonella enterica* serotype *typhi* (*S. typhi*) causes a serious systemic disease known as typhoid fever, and is responsible for up to 600,000 deaths annually, especially in southeast Asia (Crump and Mintz, 2010). In contrast, non-Typhoidal *S. enterica* serotypes such as Typhimurium and Enteritidis (*Salmonella typhimurium* and *Salmonella enteritidis*) usually cause a self-limiting inflammatory diarrhoeal disease in immunocompetent humans (Rivera-Chavez and Baumler, 2015). *Salmonella* gastroenteritis (caused by these and other serotypes) is also a significant cause of mortality and is thought to be responsible for around 155,000 deaths annually (Majowicz et al., 2010). In immunocompromised or malnourished individuals, in particular in sub-Saharan Africa, non-Typhoidal *Salmonella* infection can manifest as an invasive disease, characterized by bacteraemia and a high mortality rate, made more serious by increasing rates of antibiotic resistance (Feasey et al., 2012; Uche et al., 2017). Despite, these differences in disease outcome, all *Salmonella* serotypes must overcome the same initial barrier to infection. Following ingestion and passage through the stomach, *Salmonella* must cross the intestinal epithelium in order to successfully colonize the host.

**Figure 1:**
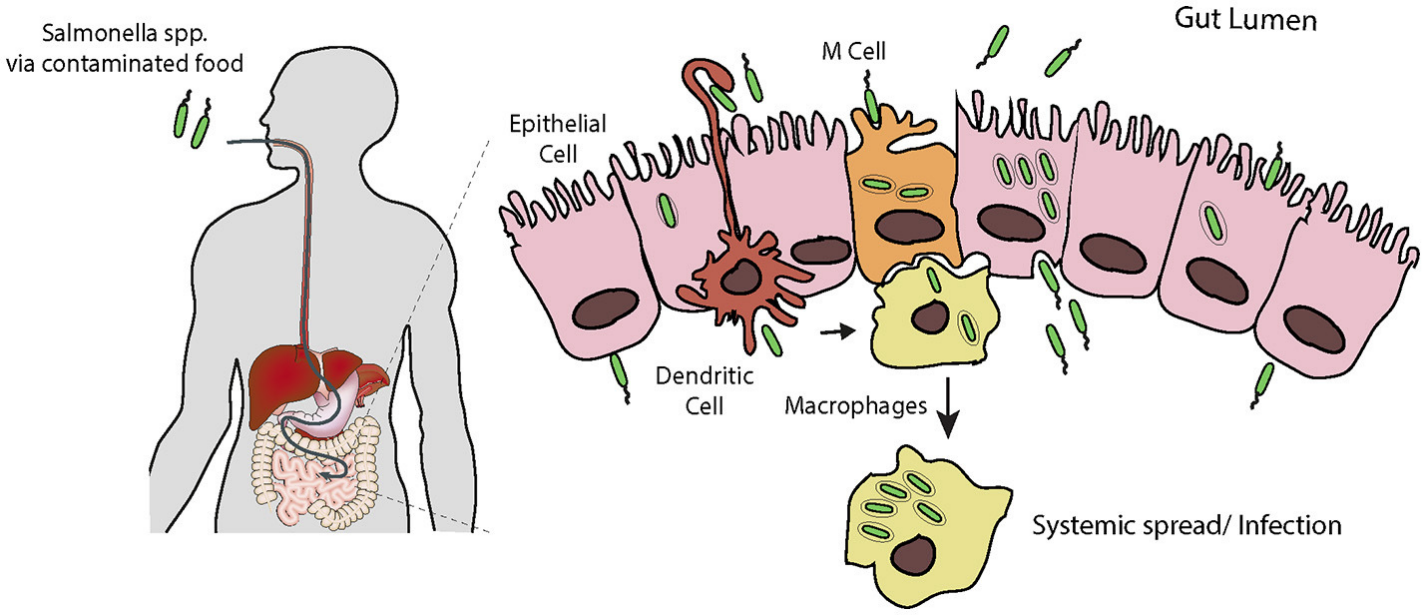
*Salmonella* infection requires invasion of host cells. Following ingestion and passage through the stomach, *Salmonella* encounters the intestinal epithelial layer, a barrier which must be breached. It is thought that the pathogen preferentially enters M cells, and is trancytosed and passed to underlying macrophages. Here *Salmonella* can evade killing by causing apoptosis leading to release, or survive and replicate long enough to allow systemic spread through the reticuloendothelial system. *Salmonella* can also force its own uptake by epithelial cells from the apical side, i.e., the lumen of the intestine. Following uptake, the bacteria can replicate intracellularly, or be transcytosed and released on the basolateral side, from here they can infect further epithelial cells. Additionally, dendritic cells can directly capture *Salmonella* from the lumen and transport them across the epithelium.

*S. typhimurium* exploits phagocytic intestinal cells, such as antigen-sampling M cells and dendritic cells, but also forces its own uptake into non-phagocytic epithelial cells (Figure 1; Santos and Baumler, 2004; Tahoun et al., 2012). Once across the epithelium, *S. typhimurium* can efficiently invade further epithelial cells from the basolateral side (Criss and Casanova, 2003), where the pathogen can survive and replicate. *S. typhimurium* usually remain localized to intestinal tissues, where the host's inflammatory response to the invading pathogen is responsible for the symptoms of gastroenteritis (Zhang et al., 2003). It has recently become apparent that in cases of *Salmonella* gastroenteritis, only a small proportion of the ingested bacteria invade the epithelium in this manner. Instead the bulk of the population remain in the lumen of the intestine, where they gain a selective advantage over the resident microbiota due to the host's inflammatory response to the small invading sub-population (Stecher et al., 2007; Raffatellu et al., 2009; Winter et al., 2010). Although, *S. typhimurium* can survive and replicate within macrophages, the invading sub-population will eventually be cleared, likely by host neutrophils. However, when this fails, e.g., in the immunocompromised, systemic spread, and bacteraemia can occur (Gordon, 2008).

*S. typhi* enters and crosses the intestinal epithelium in an analogous manner to *S. typhimurium*, however largely due to its polysaccharide capsule this does not trigger an inflammatory response (Wangdi et al., 2012; Keestra-Gounder et al., 2015). The invading pathogen can also survive and replicate in macrophages, which allows systemic spread. *S. typhi* colonizes host organs such as the spleen, liver, and gallbladder, inducing the symptoms of typhoid fever (Dougan and Baker, 2014). Studies using the mouse model of typhoid fever (i.e., infection of mice with *S. typhimurium*) have identified phagocytic cells such as macrophages, and later PMNs and mononuclear cells, as the predominant cell type infected during the systemic phase of disease (Mastroeni and Grant, 2013), though it remains to be proven if this holds true for *S. typhi* infection in humans. The role of entry into extra-intestinal epithelial cells is uncertain, but may be especially important in the gallbladder, where SPI1-dependent entry could allow chronic infection and consequent long term shedding (Gonzalez-Escobedo and Gunn, 2013).

It is clear that the ability to enter host cells is fundamental to *Salmonella* pathogenesis. It is therefore perhaps not surprising that the pathogen has evolved multiple seemingly redundant mechanisms to achieve this in a range of host cell types. While some of these pathways have been the subjects of intense research, others are only beginning to be identified. The aim of this review is to summarize our understanding of how *Salmonella* promotes its own uptake by non-phagocytic cells, with a focus on the host cell signaling pathways that are subverted.

## The canonical view—*Salmonella* pathogenicity island 1 entry effectors

Almost 30 years ago, genes responsible for the ability of *Salmonella* to enter non-phagocytic cultured cells were identified (Galan and Curtiss, 1989), and shown to be part of a pathogenicity island termed SPI1 (*Salmonella* Pathogenicity Island 1; Collazo and Galan, 1997b). SPI1 has been the subject of decades of research, which has shown it to encode a Type Three Secretion System (T3SS), a molecule syringe that directly delivers a cohort of virulence effector proteins (encoded both within SPI1 and elsewhere in the *Salmonella* chromosome) into host cells (Deng et al., 2017). These effectors both drive the forced uptake of the pathogen by non-phagocytic cells, and also manipulate host cell signaling pathways, especially those involved in the inflammatory response (McGhie et al., 2009). While the biochemical activities of the entry effectors are well-characterized, their contributions to the invasion process are multi-faceted and only beginning to be fully understood.

The paradigm of *Salmonella* forced uptake is that the SPI1-delivered effectors induce rearrangements of the host cell actin cytoskeleton, leading to the production of large lamellipodia-like surface protrusions termed membrane ruffles, which eventually engulf the pathogen in a process similar to macropinocytosis, the uptake of large volumes of extracellular solutes and nutrients by cells (Garcia-del Portillo and Finlay, 1994). Mutagenesis studies have identified the effectors responsible for ruffling, and therefore entry.

SopB, SopE, and SopE2 have been identified as the major drivers of uptake, as strains in which all three genes are mutated are virtually non-invasive (Zhou et al., 2001). SopB is a lipid phosphatase which can remove phosphates from the 4′ and 5′ position of various phosphatidylinositol species, and both of these activities are required for SopB-mediated ruffle formation (Piscatelli et al., 2016). Infection of cultured cells leads to a SopB-dependent increase in phosphatidylinositol-3-phosphate (PI3P), phosphatidylinositol-3,4-bisphosphate (PI(3,4)P_2_)and phosphatidylinositol-3,4,5-triphosphate (PI(3,4,5)P_3_) (Mallo et al., 2008). The production of these lipids allows the recruitment of various host proteins, e.g., SGEF, which in turn activates RhoG, which may be important for *Salmonella* entry (Patel and Galan, 2006). The signaling cascades triggered by these phosphatidylinositol species are described in later sections.

The precise mechanism by which the lipid phosphatase SopB leads to increased levels of these phosphoinostide species remains uncertain. SopB activity is required for Rab5 recruitment to the SCV. Vps34, a PI3-kinase, then associates with active Rab5, and is thought to be responsible for PI3P formation on SCVs (Mallo et al., 2008). SopB manipulation of phosphoinositide dynamics at the plasma membrane is more complex. In cells expressing SopB, PI(4,5)P_2_ in the plasma membrane is dephosphorylated (Terebiznik et al., 2002), generating PI4P and PI5P. It has been proposed that this increase in PI5P may activate Class II PI3-kinases, which leads to the production of PI(3,4)P_2_ and PI(3,4,5)P_3_ (Mallo et al., 2008), and direct hydrolysis of these by SopB may produce PI3P at membrane ruffles (Piscatelli et al., 2016). It is therefore likely that SopB controls dynamic cycles of phosphoinositide production at sites of *Salmonella* entry (Piscatelli et al., 2016).

SopE2 is encoded by essentially all *Salmonella* strains, and is a mimic of host guanine nucleotide exchange factors (GEFs) that activates the Rho family GTPase Cdc42 (Stender et al., 2000). In addition to SopE2, a number of strains have been lysogenized with a bacteriophage which encodes SopE (Mirold et al., 1999), a second GEF mimic, highly similar to SopE2. SopE activates Cdc42 and also Rac1 (Hardt et al., 1998a; Friebel et al., 2001), which as discussed below is crucial for triggering membrane ruffling and pathogen uptake, and deletion of *sopE* in strains that encode it reduces invasion of cultured cells by around 50% (Hardt et al., 1998b). However, Rac1 activity is also central to promoting the inflammatory response associated with *Salmonella* infection, and it has been proposed that this activity, along with the selective advantage it gives *Salmonella* in the lumen of the inflamed intestine in gastroenteritis, is the evolutionary driver for SopE acquisition (Lopez et al., 2012). The cellular consequences of SopE/E2 activation of Cdc42 and Rac1 are discussed further below.

In addition to these effectors which indirectly target the actin cytoskeleton by manipulating signaling networks, the SPI1 effectors SipA and SipC are direct actin-binding proteins, and consequently prime candidates for promoting pathogen uptake (Hayward and Koronakis, 2002). SipC both nucleates actin filament polymerization and bundles pre-existing actin filaments (Hayward and Koronakis, 1999). Studying the role of SipC-mediated actin assembly in *Salmonella* entry is complicated due to SipC being a key component of the SPI1 T3SS translocon, the pore-like structure that inserts in the host cell plasma membrane and allows the translocation of effectors into the target cell. SipC null mutants strains of *Salmonella* are non-invasive (Kaniga et al., 1995), but also defective in delivery of any effector proteins (Collazo and Galan, 1997a). Attempts have been made to separate the two activities of SipC by making a series of small internal deletions in the protein. One such mutant is capable of restoring effector delivery when expressed in a *sipC* deletion strain, but does not fully complement the invasion defect, suggesting SipC is indeed directly involved in promoting entry (Chang et al., 2005; Myeni and Zhou, 2010).

SipA potentiates the actin nucleating and bundling activities of SipC (and also the host bundling protein T-plastin; McGhie et al., 2001). SipA binds with high affinity and stabilizes actin filaments, both mechanically and by preventing their depolymerization by host proteins such as ADF and cofilin (McGhie et al., 2004). SipA mutants have small, but significant, decrease in invasiveness, suggesting that SipA enhances *Salmonella* entry but is not strictly required (Jepson et al., 2001).

## Cellular pathways targeted by *Salmonella* entry effectors

The direct cellular targets of the *Salmonella* entry effectors (e.g., Rho GTPases, phosphoinositide lipids) are master regulators of numerous divergent signaling networks. Consequently, the injection of these effectors into targets cells triggers many different pathways, often with overlapping outcomes. While these signaling pathways influence multiple aspects of *Salmonella* pathogenesis, the focus here will be those that have been shown to drive pathogen uptake.

### The wave regulatory complex

**Figure 2:**
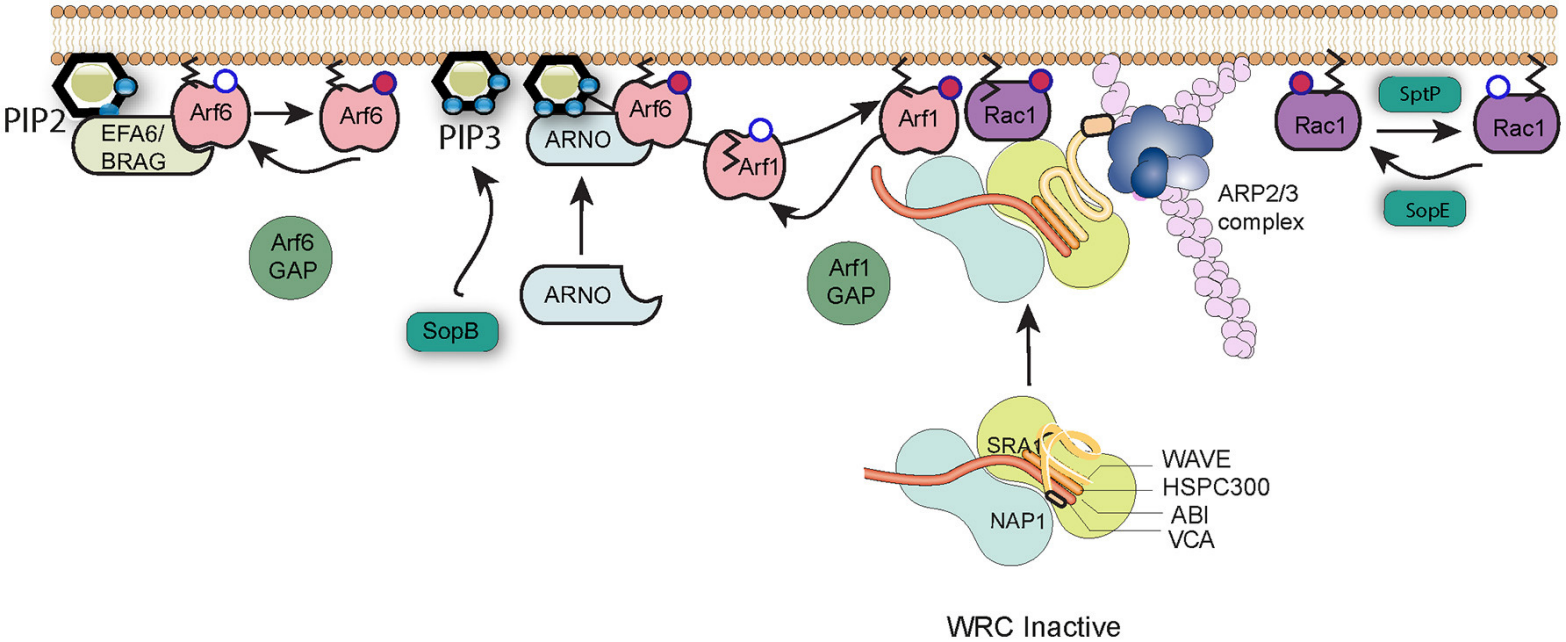
*Salmonella* subversion of the WAVE regulatory complex. The Wave regulatory complex (WRC) exists in an inactive state. Upon effector protein delivery by *Salmonella*, Arf6 is activated by host cell GEFs such as EFA6 and BRAG, which stimulate the exchange of GDP (white circle) bound to Arf6 for GTP (red circle). Active Arf6, along with the lipid PI(3,4,5)P_3_ generated by SopB, recruits the host GEF ARNO that in turn activates Arf1. Arf1 consequently anchors via its exposed myristoylation moiety (black lines) to the plasma membrane, where active Arf1 and SopE-activated Rac1 work in cooperation to recruit and activate the WRC and induce Arp2/3-dependent polymerization of actin filaments (pink). Arf1 can subsequently be inactivated by cellular GAPs, and cycles of activation and inactivation promote invasion efficiency. Signaling can be switched off by SptP-mediated inactivation of Rac1.

Early studies of *Salmonella* entry showed that the membrane ruffles that are generated by the pathogen morphologically resemble lamellipodia, flat sheet-like extensions of the plasma membrane that the cell uses to adhere to and move over surfaces (Takeuchi, 1967). Lamellipodia formation requires the host WAVE Regulatory Complex (WRC; Bisi et al., 2013), a heteropentameric complex composed of WAVE (WASP family verprolin homolog), Abi (abl-interactor), Cyfip (cytoplasmic FMR1 interacting protein), Nap1 (NCK-associated protein 1), and HSPC300 (heat shock protein C300), or their homologs (Chen et al., 2010). WAVE is a nucleation promoting factor (NPF), an activator of the Arp2/3 complex, the cells major actin nucleation machinery, which in turn drives the formation of branched networks of actin filaments that form the lamellipodium (Bisi et al., 2013). It is well-established that the same pathway promotes the pronounced membrane ruffling associated with *Salmonella* entry (Hanisch et al., 2010), and recent work has identified the multiplicity of signaling events that must be coordinated by *Salmonella* in order to correctly subvert WRC function (Figure 2).

The activity of the WRC is governed by a remarkable interplay between Rac1 and Arf1 GTPases (Singh et al., 2017), which are both required to recruit the WRC to the membrane and promote subsequent Arp2/3 activation (Koronakis et al., 2011). As described above, the *Salmonella* entry effector SopE is a GEF mimic which directly activates Rac1, a process that possibly requires the host membrane protein caveolin (Lim et al., 2014). However, *Salmonella* does not encode any known Arf GEFs, and instead must exploit host regulatory networks to achieve the Arf1 activation necessary for WRC activation (Humphreys et al., 2012). Arf1 is best known for its activities in membrane trafficking at the Golgi membrane, but can be recruited to the plasma membrane by the cellular GEF ARNO (Arf nucleotide-binding-site opener). ARNO is maintained in the cytosol in an autoinhibited conformation but is recruited and activated at the plasma membrane via GTP-bound Arf6 and acidic phospholipids such as PI(3,4,5)P_3_ that interact with the protein's Pleckstrin Homology (PH) domain (Cohen et al., 2007). The production of PI(3,4,5)P_3_ at sites of *Salmonella* internalization is triggered by SopB, but Arf6 is activated by the cellular GEFs BRAG and EFA6 (Humphreys et al., 2013). These host factors are known to be activated at least in part through interaction with PI(4,5)P_2_, though the means by which they are specifically recruited and activated by the pathogen has yet to be uncovered.

GTPases such as Rac1 and Arf1 are turned off by proteins called GAPs, GTPase activating proteins, which trigger hydrolysis of bound GTP to GDP. *Salmonella* encodes its own Rac1 GAP, SptP, which following pathogen uptake, returns the cell's signaling to the resting state (Fu and Galan, 1999). As is the case for the GEFs, *Salmonella* encodes no known Arf GAP, but instead hijacks host proteins (Davidson et al., 2015). Remarkably however, the function of these extends beyond simply turning off signaling. When the expression of Arf6 GAPs ACAP1 and ADAP1 or the Arf1 GAP ASAP1 was knocked down by RNAi, entry of *Salmonella* into cultured cells was significantly impaired (Davidson et al., 2015). This suggests that WRC-driven actin assembly requires cycles of Arf activation and inactivation. *Salmonella*-induced membrane ruffling thus represents a fascinating interplay between Arf and Rac1 GTPases, between host and pathogen regulatory proteins, and between positive and negative control mechanisms.

### The WASH complex

*Salmonella*-induced membrane ruffling has long been thought of as the driving force behind internalization, but in recent years it has become apparent that the two activities can be separable, at least in certain cell types. In cultured fibroblasts, if components of the WRC are depleted by RNAi, membrane ruffles are completely abolished but pathogen uptake can still be observed (Hanisch et al., 2010). This residual entry could be further reduced by additional Arp2/3 knockdown, suggesting that an NPF other than WAVE must be responsible for this activity. Surprisingly this NPF is not NWASP, which is activated by Cdc42 and was previously thought to be required for *Salmonella* invasion. In fact, an NWASP knockout cell line showed increased levels of pathogen entry (Hanisch et al., 2010), A screen of other known NPFs identified a role for the WASH complex (Hanisch et al., 2010), a heteropentamer with a similar architecture to the WRC, composed of the NPF WASH (Wiskott-aldrich syndrome protein and scar homolog), Strumpelin, SWIP (Strumpelin and WASH interacting protein), FAM21 (Family number 21, also known as Vaccina Penetration Factor or VPEF), and CCDC53 (coiled-coil domain containing protein 53).

**Figure 3:**
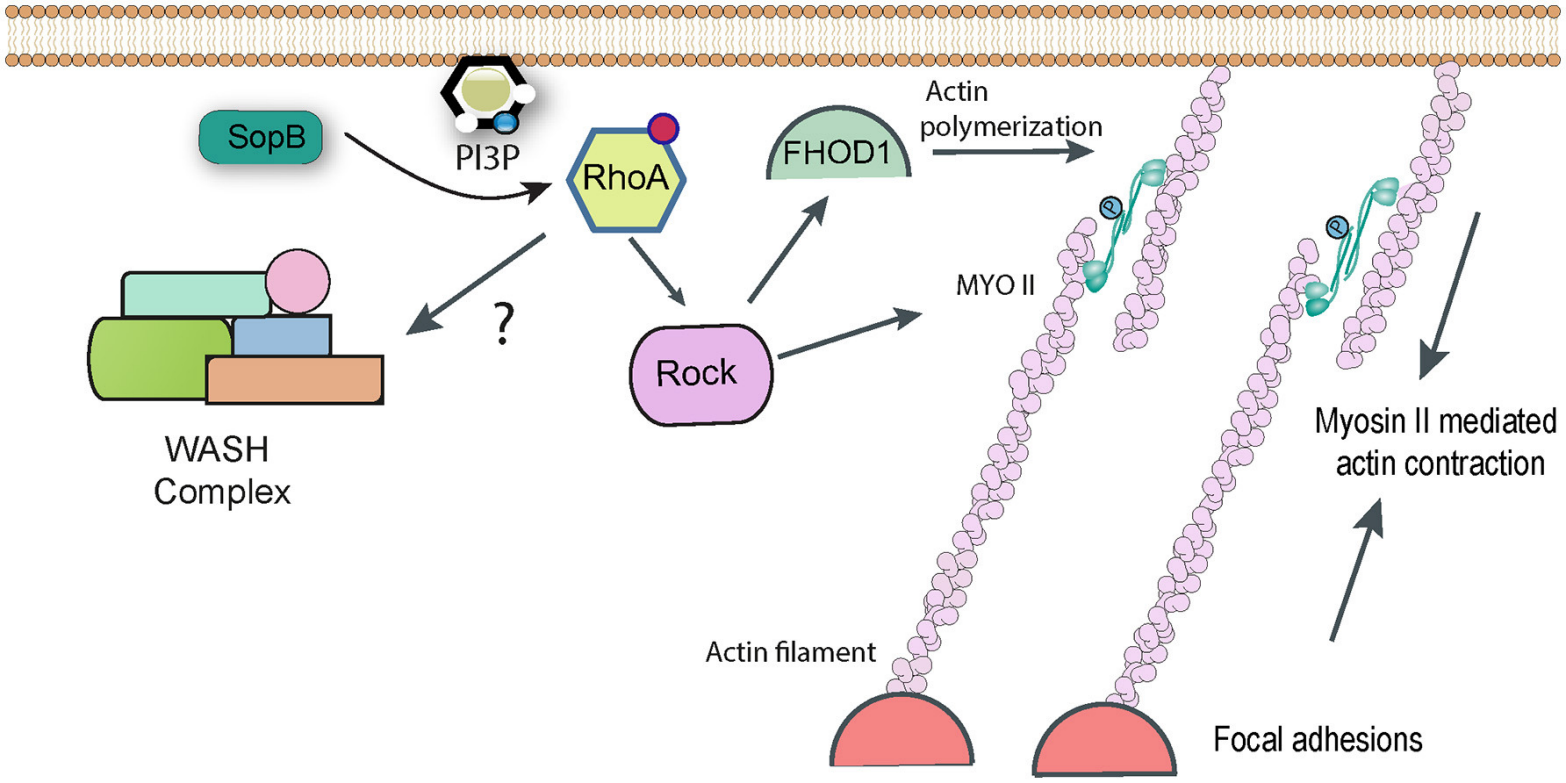
RhoA-dependent pathways targeted by *Salmonella*. Following delivery of SopB into host cells RhoA becomes activated by an unknown mechanism, presumably involving the generation of phopshatidylinositol lipids. RhoA can activate Rho kinase (ROCK), which in turn may be responsible for activating Myosin II-mediated contractility, which contributes to *Salmonella* uptake. Rock may also activate the formin FHOD1, which can directly polymerize actin filaments. In addition, it has been reported that RhoA can directly activate the WASH complex, to promote Arp2/3-dependent actin assembly, though this link remains hypothetical.

The WASH complex was only discovered fairly recently (Linardopoulou et al., 2007; Derivery et al., 2009; Gomez and Billadeau, 2009; Jia et al., 2010) and little is known about its regulation in the cell. Its main function seems to be in promoting Arp2/3-dependent actin polymerization on endosomes and lysosomes. The WASH complex was however shown to accumulate at sites of *Salmonella* entry into fibroblasts, and knockdown of complex components resulted in a small, though statistically significant, impairment of pathogen uptake (Hanisch et al., 2010). The WASH complex accumulates on phagosomes and macropinosomes in *Dictyostelium*, where it plays a key role in recycling certain proteins to the plasma membrane and avoiding their degradation (Buckley et al., 2016). It remains to be determined whether WASH has a similar function during *Salmonella* uptake, or whether it plays a more direct role in actually driving actin-dependent pathogen entry. The *Salmonella* effectors responsible for subverting WASH have yet to be identified. It has been reported that WASH acts downstream of Rho1 (Liu et al., 2009), the *Drosophila* ortholog of mammalian RhoA. The *Salmonella* effector SopB can indirectly trigger RhoA activation (Figure 3; see below), but any link between SopB and WASH has not been reported.

### Myosin-mediated contractility

While Arp2/3 is the cell's best-characterized nucleator of actin polymerization, other pathways do exist. When expression of Arp2/3 is knocked down in cultured fibroblasts, *Salmonella* invasion is severely reduced but is not completely abolished (Hanisch et al., 2010). This suggests the existence of Arp2/3 independent entry pathways. It was noticed that *Salmonella* infection of fibroblasts often led to the generation of actin stress fibers in the cells, which were specifically decorated with myosins IIA and IIB (Hanisch et al., 2011). Myosins are molecular motors and ATP hydrolysis by myosins can cause them to slide along and generate contractive forces on anchored actin filaments. When cells were treated with chemical inhibitors of mysoin II, pathogen uptake was reduced by around 50% (Hanisch et al., 2011). Importantly, this inhibition was additive with Arp2/3 RNAi, confirming that the two represent separate pathways of bacterial entry.

Entry of Δ*sopB Salmonella* (assumed to be driven in the main by SopE/E2) was unaffected by myosin inhibition, whereas Δ*sopE/E2* strains (in which it is assumed that SopB is responsible for uptake) were efficiently inhibited, suggesting that SopB is the effector responsible for hijacking myosin II (Hanisch et al., 2011). The GTPase RhoA and its downstream target Rho Kinase are known activators of myosin II, and indeed chemical inhibition of either gave a similar phenotype to myosin inhibition for SopB-mediated uptake (Hanisch et al., 2011). Consequently a pathway can be drawn in which SopB activates RhoA, this activates Rho Kinase which in turn phosphorylates and activates myosin II-dependent contractility, allowing it to contribute to *Salmonella* uptake (Figure 3). The molecular detail of how the lipid phosphatase SopB activates RhoA remain to be fully determined. The products of cellular phosphatidylinositol-3-kinase (PI3K), such as PI(3,4)P_2_ and PI(3,4,5)P_3_, are proposed to act upstream of RhoA activation. Despite being a phosphatase that cleaves phosphate from both the 4′ and 5′ position, both PI(3,4)P_2_ and PI(3,4,5)P_3_ (along with PI3P) accumulate in infected cells in a SopB-dependent manner (Mallo et al., 2008), which could explain the activation of the RhoA pathway.

### Formins

Formins are a family of proteins that control cell shape, adhesion, and cytokinesis by promoting actin polymerization independently of Arp2/3. The existence of Arp2/3-independent entry pathways for *Salmonella* made the formin family an obvious target for investigation, and indeed studies with chemical inhibitors and RNAi identified the formin FHOD1 as playing a role in pathogen uptake (Truong et al., 2013). When FHOD1 is knocked down, *Salmonella* ruffles are much smaller, whereas Arp2/3 knockdown leads to the production of long filopodia-like extensions rather than the typical lamellipodia-like structures. It has been suggested that FHOD1 may generate new actin filaments early in the entry process, which Arp2/3 can then bind to and generate the branched actin network required for broad ruffle formation. Consistent with this, FHOD1 is recruited to *Salmonella* entry sites before Arp2/3 (Truong et al., 2013).

Interestingly, as with myosin II, FHOD1 lies downstream of RhoA and Rho Kinase in signaling pathways, suggesting that SopB is likely responsible for targeting the formin (Figure 3). Consistent with this, Δ*sopB Salmonella* showed reduced activation of FHOD1 (Truong et al., 2013). However, a similar phenotype was observed with a Δ*sopE*/*E2* strain (Truong et al., 2013). Early studies suggested that the GEF SopE could activate RhoA *in vitro* (Hardt et al., 1998a), though this is not thought to happen during *Salmonella* invasion (Criss and Casanova, 2003). It has been suggested that FHOD1 can also be directly activated by Rac1 (Westendorf, 2001), which can certainly be activated by SopE (Patel and Galan, 2006). Consequently, the *Salmonella* effectors and the precise pathway that regulates FHOD1 remains to be determined conclusively.

FHOD1 is not the only non-Arp2/3 actin nucleator implicated in *Salmonella* entry. A recent genome-wide siRNA screen identified a role for SPIRE1 and 2 (Andritschke et al., 2016), proteins which nucleate the assembly of straight actin filaments. However, the nature of the involvement of SPIRE remains somewhat uncertain. The two isoforms seem to act at different stages of pathogen entry, with SPIRE1 effecting the initial pathogen binding event and SPIRE2 playing more of a role in establishing a replicative niche following entry (Andritschke et al., 2016). An “effectorless” *Salmonella* strain complemented only with either SipA (which invades cells without triggering ruffling) or with SopE (which promotes profuse ruffles) were each impaired equivalently by SPIRE knockdown. In fact, a similar phenotype was still observed in a SPI1 T3SS mutant strain in which uptake is driven by expression of the *Yersinia* Invasin gene (Andritschke et al., 2016), which triggers uptake by the “zipper” mechanism mediated by interaction with α5β1 integrins. Therefore, impact of SPIRE knockdown on bacterial uptake is independent of the mechanism of entry, and it is unclear whether SPIRE actually plays a direct role in the actin assembly that drives the internalization of *Salmonella*, or whether it has a more indirect function.

### The exocyst complex

**Figure 4:**
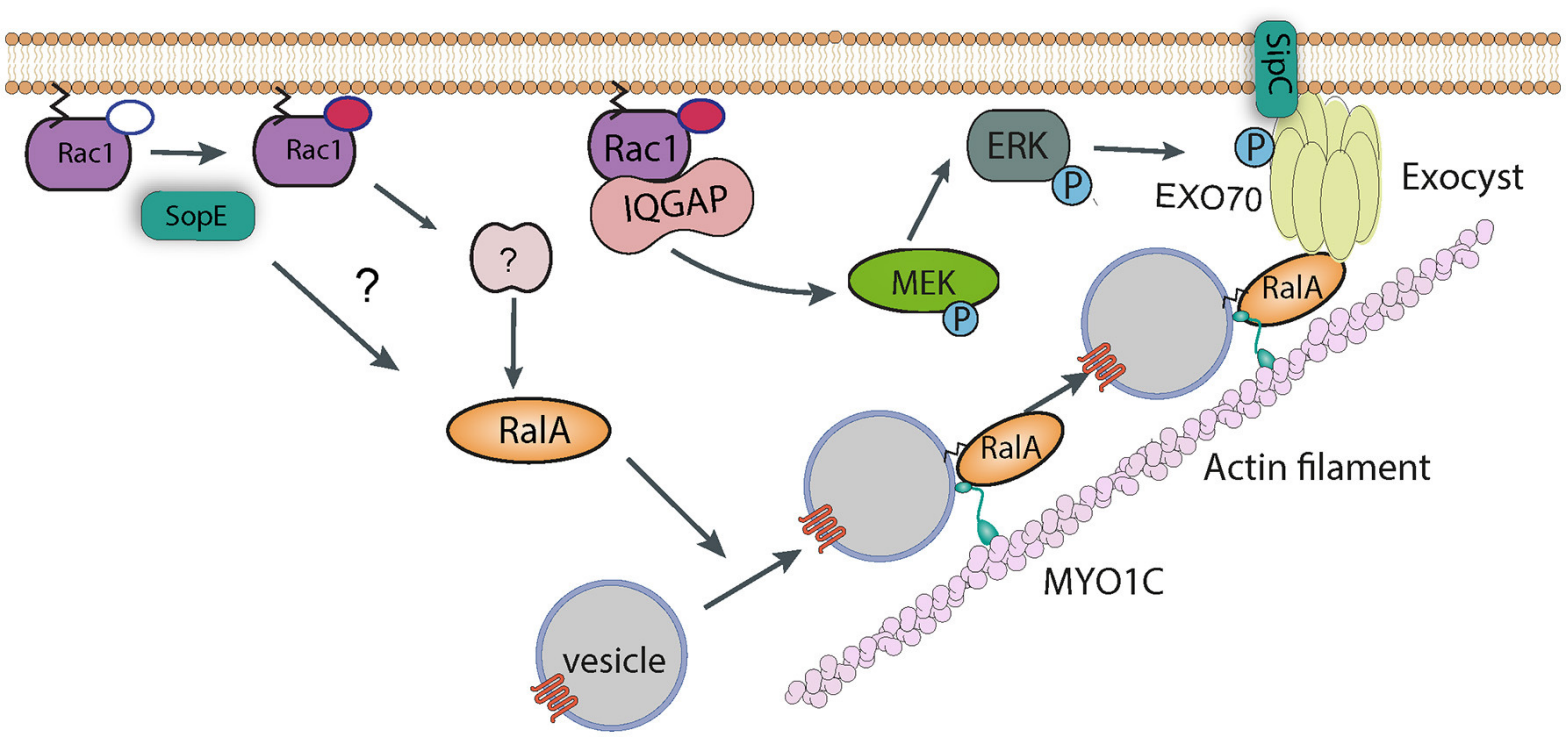
Manipulation of vesicle trafficking by *Salmonella*. Injection of SopE leads to activation of RalA, either directly or via Rac1 and some unknown host factor. RalA may bind to both Myosin 1c and the octomeric exocyst complex, which cooperate to target vesicles to sites of *Salmonella* entry. SipC also contributes to the recruitment of the exocyst complex by directly binding Exo70. Rac1, activated by SopE, is maintained in the GTP-bound state by interaction with IQGAP1, which also acts as a scaffold and recruits the kinase MEK. It is possible that MEK activates ERK, which in turn phosphorylates Exo70, promoting exocyst complex assembly.

In addition to directly nucleating actin polymerization and bundling actin filaments, a yeast two-hybrid screen identified Exo70 as a potential binding partner of the SipC C-terminal domain (Nichols and Casanova, 2010). Exo70 is part of the hetero-octameric exocyst complex, the machinery that targets vesicles to the plasma membrane prior to their fusion. The exocyst complex was shown to accumulate at sites of *Salmonella* invasion in cultured cells, and knockdown of exocyst components led to a modest, though significant, decrease in internalization (Nichols and Casanova, 2010). Assembly and recruitment of the exocyst complex is known to require RalA, a small GTPase (Nichols and Casanova, 2010). Importantly, and consistent with a role for this complex in invasion, *Salmonella* activates RalA. The effector SopE is responsible for activating RalA, with a possible contribution from SopE2, though it remains uncertain whether this is a result of direct GEF activity for RalA or, perhaps more likely, an indirect pathway involving a cellular RalA GEF (Figure 4). Consistent with this hypothesis, transfection of cultured myoblasts with constitutively-active Rac1 (i.e., the confirmed substrate for SopE) leads to activation and membrane recruitment of RalA (Nichols and Casanova, 2010).

The precise role of exocyst recruitment in *Salmonella* uptake remains to be confirmed. The canonical internalization pathway induces pronounced membrane ruffles to drive pathogen uptake. A long-standing question has been whether the cell's membrane is truly flexible enough to form these long protrusions without extra phospholipids being incorporated. This would be a particular problem for cells that internalize multiple bacteria, as phospholipids are removed from the membrane as SCVs form. The suggestion that *Salmonella* may exploit the exocyst complex to promote fusion of vesicles at sites of internalization would provide a source of extra phospholipids. Interestingly, when expression of exocyst components is knocked down, *Salmonella* induces much smaller membrane ruffles (Nichols and Casanova, 2010). However, the exocyst complex has been reported to directly bind and recruit the WRC (Biondini et al., 2016), suggesting that its role could be in delivering not only lipids but also the protein responsible for membrane ruffling. The situation is further complicated by the observation that the exocyst can directly bind and activate Arp2/3 (Zuo et al., 2006), suggesting it could actually play a more direct role in the cytoskeletal rearrangements underlying pathogen uptake. The precise reason for *Salmonella* recruiting the exocyst complex remains to be determined. However, it is clear that in the cell, vesicle trafficking and membrane-localized cytoskeletal reorganization are intimately linked, a fact that a specialized invasive pathogen such as *Salmonella* must have evolved to exploit.

In support of this, the actin motor protein Myosin 1c has also been shown to be important for delivering signaling components to the plasma membrane for pathogen entry (Figure 4). Cholesterol-rich membrane microdomains termed lipid rafts are enriched in signaling proteins, and can be internalized and stored in the perinuclear region of the cell. Myosin 1c is required to recycle these lipid rafts back to the plasma membrane. When this pathway is blocked by Myosin 1c RNAi, *Salmonella* entry is reduced (Brandstaetter et al., 2012). The role of specific effectors in subverting Myosin 1c have not been investigated, however interestingly, RalA is known to bind to Myosin1c (Chen et al., 2007), suggesting a synergy with the exocyst complex. It is thus possible that Myosin 1c is required for the delivery of raft-containing vesicles to the plasma membrane, where the exocyst complex drives fusion between these vesicles and the membrane.

### IQGAP1

IQGAP1 is a protein which consists of multiple domains, including several calmodulin-binding IQ domains and a domain with homology to GTPase-activating proteins (GAPs, inactivators of Rho GTPases), which give the protein its name (Watanabe et al., 2015). IQGAP1 has been shown to localize to *Salmonella* entry sites, probably via its actin binding domain, and its knockdown or knockout reduces invasion significantly (Brown et al., 2007). The putative GAP domain is actually a Rho GTPase binding domain which maintains these GTPases in an active, GTP-bound state, and in its absence active Rac1 and Cdc42 no longer accumulate in infected cells (Brown et al., 2007), suggesting that following activation by SopE/E2, IQGAP1 is required to protect GTPases from inactivation.

In addition to this activity, IQGAP1 has also been proposed to act as a molecular scaffold, and binds directly to various proteins including MEK (MAPK/ERK kinase). *Salmonella* invasion of IQGAP1 knockout cells complemented with a Rac1/Cdc42-binding, or MEK-binding mutant of IQGAP1 was partially restored in both cases (Kim et al., 2011). In addition, entry into knockout cells complemented with the Rac1/Cdc42-binding mutant could be completely abrogated by a MEK inhibitor (Kim et al., 2011). This all suggests that the MEK actually plays an important role in the entry process. Interestingly, one of the substrates of MEK is ERK1/2 (extracellular signal-regulated kinase 1 and 2), which itself has been shown to phosphorylate Exo70 and promote assembly of the exocyst complex (Ren and Guo, 2012). In addition, IQGAP1 has been proposed to directly bind and regulate the function of the exocyst complex (Sakurai-Yageta et al., 2008). It is tempting to speculate that IQGAP1 contributes to *Salmonella* invasion by subverting membrane traffic in this way (Figure 4), though this has not been tested experimentally.

It is worth noting that IQGAP1 can also act as a scaffold for various phosphoinositide kinases (Choi et al., 2016). Such complexes can contain phosphatidylinositol 4-kinase III α (PI4Kα), phosphatidylinositol phosphate kinase I α (PIPKIα) and phosphatidylinositol 3-kinase (PI3K), i.e., all of the enzymes required to sequentially phosphorylate phosphatidylinositol for *de novo* generation of PI(3,4,5)P_3_ (Choi et al., 2016). IQGAP1 could thus collaborate with SopB in the generation of PI(3,4,5)P_3_ at *Salmonella* entry foci.

### Annexins

Annexins are calcium-dependent membrane binding proteins. They play a key role in membrane organization and trafficking. A number of individual annexins, such as annexins A1, A2, and A5, have been demonstrated to also bind actin, and hence function to regulate cytoskeleton-membrane dynamics. As described in the previous section, the interface between the actin cytoskeleton and the plasma membrane is of central importance to *Salmonella*'s forced internalization and is consequently a key target for injected effectors. Perhaps predictably then, annexin A2 (AnxA2) has been reported to be required for efficient *Salmonella* invasion (Jolly et al., 2014).

At the plasma membrane, AnxA2 primarily forms a heterotetramer comprised of two molecules of AnxA2 and two molecules of a protein called p11. Microscopy studies showed that both AnxA2 and p11 are specifically enriched at *Salmonella* entry ruffles in cultured cells, and RNAi-mediated knockdown of either protein significantly reduced invasion (Jolly et al., 2014). The AnxA2/p11 complex binds directly to AHNAK, a very large phosphoprotein that is thought to cooperate in regulating membrane and cytoskeletal rearrangements. AHNAK too was recruited by *Salmonella*, and its knockdown reduced uptake (Jolly et al., 2014). The effectors responsible for subverting the AnxA2/p11/AHNAK pathway remain to be conclusively demonstrated. Deletion of either SopB or SopE/E2 reduced the enrichment of these proteins at entry foci, so it may be a multifactorial process, but no direct interactions have yet been reported. AHNAK is a substrate for the kinase AKT, which is known to be activated in cells by SopB. However, AKT was not required for AHNAK recruitment by *Salmonella* (Jolly et al., 2014).

### Myosin VI

As described above, Myosin motor proteins use the energy derived from ATP hydrolysis to move cargo along actin tracks. Myosin VI is an unusual member of the Myosin family, as unlike almost all other myosins it moves toward the minus end of actin filaments (Buss et al., 2004). Myosin VI can deliver cargo to the cell surface and plays a key role in numerous cellular functions including endocytosis. A link between Myosin VI and *Salmonella* uptake was first identified from *in vitro* reconstitution of SopE signaling at model lipid membranes (Brooks et al., 2017). SopE was shown to specifically recruit Myosin VI (along with other proteins) to these lipid bilayers. SopE activates the GTPase Rac1 to trigger WRC-dependent actin polymerization (Humphreys et al., 2012), and this assembly of actin filaments was required for Myosin VI recruitment (Brooks et al., 2017). Rac1 also has other downstream signaling targets in the cell, one of which is p21 activated Kinase (PAK). Interestingly, Rac1-induced phosphorylation of Myosin VI by PAK was also necessary, showing that two distinct pathways converge to recruit Myosin VI to the membrane (Brooks et al., 2017).

**Figure 5:**
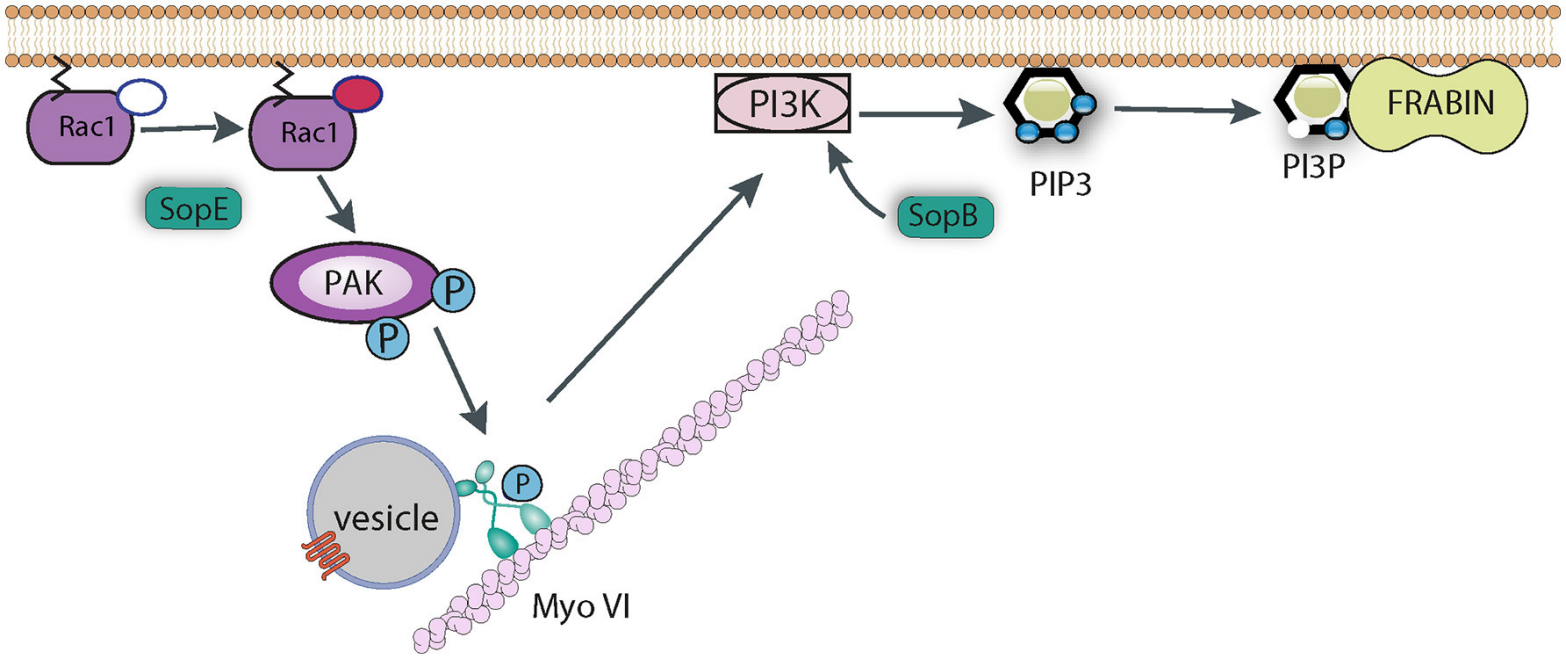
Role of Myosin VI during *Salmonella* invasion. SopE activates Rho GTPases such as Rac1, which induce the recruitment of Myosin VI (Myo VI) to the membrane via PAK-mediated phosphorylation. SopB and Myosin VI (possibly via delivering some cargo vesicle to the membrane) are required to trigger PI3K signaling to generate PIP3. SopB dephosphorylates PI(3,4,5)P_3_ to generate PI3P, which in turn recruits PI3P-binding proteins, such as Frabin, that promote *Salmonella* uptake.

Myosin VI is important for *Salmonella* uptake as its knockdown reduced invasion efficiency, and its role seems to revolve around the generation of the correct phospholipid signaling platform at the site of pathogen attachment (Figure 5). As described above, SopB is responsible for generating an enrichment of PI3P, PI(3,4)P_2_, and PI(3,4,5)P_3_, and this activity also requires a cellular PI3K (Mallo et al., 2008), Myosin VI is required for the recruitment of PI3K to the membrane, which allows SopB to generate this lipid platform, which presumably recruits a plethora of signaling protein required for membrane ruffling and pathogen uptake (Brooks et al., 2017). One such protein that has been identified is Frabin (FGD4; FYVE, RhoGEF, and PH domain-containing protein 4), a PI3P -binding, and actin-binding, GEF for Cdc42 (Ono et al., 2000). Frabin was enriched at *Salmonella* entry ruffles, and its knockdown reduced invasion by around 40% (Brooks et al., 2017). It remains to be seen which other proteins are recruited by *Salmonella* via the Myosin VI pathway. For example Sorting Nexin 9 (Snx9) has been reported to be recruited by SopB-generated PI(3,4)P_2_, and Snx9 knockdown significantly reduced entry into cultured cells (Piscatelli et al., 2016), though the role of Myosin VI upstream of Snx9 recruitment has not been investigated.

### Villin

Simple cell culture models using non-polarized cells such as HeLa have been invaluable in identifying pathways exploited by *Salmonella*, but they may inevitably misrepresent what happens in the polarized cells of the intestinal epithelium. An example of a protein required specifically for apical entry into polarized cells is villin, and actin-binding and -severing protein that is only expressed and correctly localized in differentiated intestinal epithelial cells. Knockdown of villin leads to aberrant membrane ruffle morphology, characterized by greater ruffle diameters, and consequently causes a reduction in pathogen uptake (Lhocine et al., 2015). It is perhaps surprising that an actin severing protein is required for the correct generation of actin-driven membrane protrusions, but it has been suggested that the severing of pre-existing filaments generates an increased supply of barbed ends necessary for the new filament growth needed by *Salmonella*.

Logically, the activities of villin must be tightly controlled to prevent the severing of the new filaments induced by the invading pathogen, and in fact two *Salmonella* effectors have been shown to perform such a role (Lhocine et al., 2015). SipA binds directly to actin filaments, and when bound can prevent the severing activity of villin. In addition, phosphorylation has been reported to be required for villin's activity. A rapid increase in villin phosphorylation is seen upon *Salmonella* infection, however this is quickly reversed by the effector SptP, a tyrosine phosphatase. Thus, the activity of villin is highly regulated during *Salmonella* invasion, both spatially and temporally, to allow the highly dynamic rearrangement of the actin cytoskeleton required for correct membrane ruffle generation. Importantly, the role of villin has been confirmed in a mouse model, where villin^−/−^ mice, or mice expressing only a derivative of villin that lacks severing activity, are more resistant to infection by *Salmonella* (Lhocine et al., 2015).

## SPI1-independent invasion

The SPI1 T3SS is the major factor driving *Salmonella* invasion of cells, both *in vitro* and *in vivo*. Nonetheless, recent studies demonstrate that several strains of *Salmonella* lacking expression of the SPI1 T3SS still possess the ability to invade cells of diverse origins *in vitro*, with the relative contribution of SPI1-independent pathways varying with different cell types (Aiastui et al., 2010; Rosselin et al., 2011). In addition, it was shown that SPI1 is not required for *Salmonella* internalization into a cultured 3-dimensional model intestinal epithelium (Radtke et al., 2010). Moreover, *Salmonella senftenberg* strains lacking SP1-1 have been isolated from human clinical cases, suggests that the SPI1 is dispensable for the establishment of infection in humans by this serotype (Hu et al., 2008). Taken together, these observations indicate that SPI1-independent invasion mechanisms play an important role in *Salmonella* infection and pathogenesis. Several pathways for SPI1-independent cell entry have been identified, and these will be described below.

### Rck and PagN

Rck is an outer membrane protein encoded by the large virulence plasmid of most strains of *S. enteritidis* and *S. typhimurium*, though the *rck* gene is absent from other serotypes, e.g., *S. choleraesuis and S. gallinarum* (Heffernan et al., 1992). By using both a non-invasive *E. coli* strain overexpressing Rck and latex beads coated with recombinant Rck, it was demonstrated that Rck alone is able to induce entry by a receptor-mediated process. This mechanism promotes local actin remodeling, and weak, closely adherent membrane extensions, morphologically reminiscent of the “zipper mechanism” used by *Yersinia* species to invade non-phagocytic cells (Rosselin et al., 2010). Rck is poorly expressed in *Salmonella* sp. and deletion of the *rck* gene has no noticeable effect on the invasion of either wildtype or SPI1 mutants (Rosselin et al., 2010). The qurorm sensing molecule *N*-acyl homoserine lactone promotes expression of *rck* and results in the enhanced uptake of the bacteria (Rosselin et al., 2010). Whether rck has any effect on invasion *in vivo* remains unclear.

The minimal region of Rck necessary for uptake has been identified as residues 113–159. Recently it has been shown that when added to cells, this fragment can be co-immunoprecipitated with the Epidermal Growth Factor Receptor (EGFR), suggesting this is the receptor for Rck (Wiedemann et al., 2016). Consistent with this, over-expression of EGFR in cells enhanced Rck-mediated uptake, whereas either RNAi knockdown or chemical inhibition of EGFR reduced it. EGFR is usually only localized on the basolateral side of intestinal epithelial cells, suggesting that the Rck pathway may be important only after *Salmonella* has crossed the epithelium (Wiedemann et al., 2016). The signaling downstream of Rck activation of EGFR is fairly well-characterized (Mijouin et al., 2012; Wiedemann et al., 2012). Binding triggers autophosphorylation of certain EGFR residues, which in turn allows recruitment of cellular kinases such as c-Src, which phosphorylate further EGFR residues. This then allows recruitment of class I PI3K, which leads to activation of both Rac1 and the kinase Akt. Both Akt and Rac1 have been shown to be necessary for the Rck-induced actin rearrangements that drive uptake, via activation of Arp2/3. RNAi knockdown, dominant-negative expression and chemical inhibitors have shown that all of these components are specifically required for Rck-mediated uptake. While none of the other components of this Rck signaling cascade are involved in SPI1-promoted invasion, Rac1 is central to both pathways. As described in previous sections, Rac1 promotes SPI1-dependent membrane ruffles by activating the WRC, in conjunction with Arf1 (Humphreys et al., 2012). It remains to be seen if a similar network lies downstream of Rck.

In addition to Rck, a second outer membrane protein, PagN, has also been identified as being involved in *S. typhimurium* invasion (Lambert and Smith, 2008). The pagN gene is much more widely conserved than Rck, and deletion of *pagN* in *S. typhimurium* leads to a 3-fold decrease in invasion of enterocytes without altering cell adhesion. At the cellular level, the PagN-mediated entry process is poorly characterized. It was shown that actin polymerization is required during invasion (Lambert and Smith, 2008) and that PagN is able to interact with extracellular heparin proteoglycans (Lambert and Smith, 2009). Interestingly, *pagN* is optimally expressed in intracellular conditions, i.e., following uptake. It has been postulated that this expression pattern inside cells would prime *Salmonella* to be invasive upon release from the infected cell, but the true role in pathogenesis remains to be demonstrated. *S. typhi* also encodes PagN (also known as T2544), but deletion of the *pagN* gene had no effect on invasion of cells (Chowdhury et al., 2015).

Evidence for the role of Rck and PagN in invasion all stems from *in vitro* cell culture studies, however an additional, related outer membrane protein has been implicated in cell entry of *S. typhi*, and its requirement for pathogenesis has been confirmed in an animal model (Chowdhury et al., 2015). T2942 is related to Rck, and is sufficient to promote uptake of laboratory *E. coli*. Importantly a double *t2942* and SPI1 deletion strain is minimally invasive and pathogenic in a mouse model, but expression of T2942 alone can substantially restore both properties (Chowdhury et al., 2015), suggesting SPI1 is non-essential for *S. typhi*. Interestingly STM3031, the homolog of T2942 from *S. typhimurium*, was found be redundant for invasion (Chowdhury et al., 2015), consistent with the view that SPI1 plays a much more essential role in cell entry and pathogenesis of colitis than it does for systemic disease such as typhoid fever. However, as *S. typhi* is a strict human pathogen, it remains to be determined whether results from mouse models will hold in the true host organism.

## Indirect contributions to invasion?

The sections above describe the various mechanisms by which *Salmonella* proteins directly trigger cell invasion. However, there are also various reports of *Salmonella* genes that when deleted cause a defect in pathogen entry, but for which a direct role is either absent or unproven.

SiiE (*Salmonella* intestinal infection E) is encoded on SPI4, along with a type 1 secretion system responsible for its delivery from the bacterium. Deletion of SPI4 as a whole, or SiiE individually, reduced invasion of polarized cells (but not non-polarized cells such as HeLa; Lorkowski et al., 2014). This defect is only evident during invasion of the apical side of polarized cells. In addition, SiiE antibodies can block uptake by wild type *Salmonella*. However, although required for efficient invasion, SiiE is not sufficient, and instead requires SPI1-mediated membrane ruffling. SiiE likely acts as an adhesin, promoting the binding of *Salmonella* to the apical surface of polarized cells and enhancing the function of the SPI1 T3SS.

TolC is an outer membrane channel involved in the Type 1 export of toxins and multidrug efflux. Surprisingly, deletion of *tolC* gene causes a reduction in *Salmonella* invasion efficiency (Buckley et al., 2006). It was later shown however that the expression of SPI1 is significantly attenuated in the *tolC*-mutant strain (Webber et al., 2009), which explains the invasion deficit.

HlyE is a pore-forming toxin, best characterized in pathogenic *E*. coil (Hunt et al., 2010). A HlyE homolog is encoded on the *Salmonella* pathogenicity island SPI18 and is expressed by serotypes associated with systemic infection in humans including S. Typhi and S. Paratyphi A. A *hlyE* deletion strain of *S. typhi* invades cultured epithelial cells significantly less than wild type (Fuentes et al., 2008), however, the cellular events leading to HlyE-mediated invasion have not been characterized, and it is unclear whether HlyE can directly promote uptake or simply impacts on the efficiency of one of the other entry pathways.

The Type 6 Secretion System (T6SS) is a bacteriophage-like device for injecting protein into targets cells, and essentially all Gram-negative bacteria encode at least one such system. The primary target cells are thought to be other bacteria, and the T6SS thus represents a molecular weapon used by bacteria to eliminate competition (Cianfanelli et al., 2016). However, it is becoming increasingly apparent that certain T6SS can also be used to deliver proteins into eukaryotic cells (Hachani et al., 2016), and in several cases these are associated with pathogen invasion. *S. typhimurium* has several T6SS, and the overexpression of one component of one of these systems, ClpV, or a dominant-negative derivative of the same protein, almost abolished invasion of cultured epithelial cells (Schlieker et al., 2005). Conversely, deletion of *clpV* from *S. typhi* increased invasion (Wang et al., 2011). Again further work is required to determine whether these phenotypes are explained by changes in delivery or function of other invasion factors, or whether the T6SS itself represents a novel entry pathway.

## Conclusions

The ability to invade non-phagocytic cells is fundamental to *Salmonella* pathogenesis, and understanding its molecular basis remains of critical importance. The increasing threat of antibiotic resistance means new therapeutic intervention strategies are urgently needed. The findings summarized here are the result of decades of research from numerous labs around the world. The picture that has emerged is that *Salmonella* has evolved to target multiple parallel host signaling networks to ensure that uptake takes place efficiently. It is important to remember that most *Salmonella* serotypes are zoonotic and can infect multiple species. *Salmonella* can also target multiple cell types within a given host, and these cells may be heterogeneous with respect to e.g., metabolic status, signaling activity and cell cycle phase. It is not surprising then that this pathogen can exploit so many aspects of host cell biology. The multiplicity of invasion pathways highlights the need for careful choice of model systems to study these events. The importance of an individual pathway will strongly depend on the cell type used, and this can often explain seemingly conflicting results.

The SPI1 T3SS is not always essential for pathogenesis, and certainly SPI1-independent invasion pathways exist. However, in most *Salmonella* serotypes SPI1 is the main determinant of uptake, with the level of expression of SPI1 genes closely correlated to invasiveness. It is remarkable that so few effectors are required for *Salmonella* to take over so many signaling networks within target host cells. This seems to be achieved by targeting the cell's central signaling hubs, such as Rho family GTPases or phosphoinositide lipids, and scaffold proteins such as IQGAP1. More and more components of the complex networks of signaling factors manipulated by *Salmonella* effectors are being discovered. This process has been, and will surely continue to be, aided by high-throughput technologies such as genome-wide siRNA screening. However, understanding how all of these pathways intricately fit together will only be understood by a broad, multi-disciplinary approach, encompassing biochemistry, biophysics, cell biology, genomics and classical microbiology techniques. Although, as described here, we are starting to piece together this puzzle, it would be naïve to think we know everything about what *Salmonella* does to get inside our cells.

## Author contributions

PH wrote the draft manuscript. VS prepared the figures. VS, AD, and VK revised the manuscript critically. All authors read and approved the final version of the article.

### Conflict of interest statement

The authors declare that the research was conducted in the absence of any commercial or financial relationships that could be construed as a potential conflict of interest.
